# Outpatient occupational therapy access for rural stroke survivors: barriers as perceived by therapists

**DOI:** 10.3389/fstro.2026.1850977

**Published:** 2026-09-24

**Authors:** Emily A. Stevens, Lexie J. Pfeifer, Matt P. Malcolm

**Affiliations:** 1 Institute for Stroke and Cerebrovascular Diseases, The University of Texas Health Science Center at Houston, Houston, TX, United States; 2 Department of Occupational Therapy, Rocky Mountain University of Health Professions, Provo, UT, United States

**Keywords:** disability, rehabilitation, rural health services, rural stroke, stroke rehabilitation access

## Abstract

**Aim:**

Identify occupational therapist (OTs) perceived barriers to occupational therapy (OT) access for rural stroke survivors as well as barriers in stroke specific training of therapists.

**Methods:**

An electronic gap analysis survey was developed and distributed *via* email.

**Results:**

Twenty-six OTs responded. Responses were analyzed utilizing descriptive statistics and summarization of comments. Therapist-perceived barriers included: family/caregiver support, distance to clinic, insurance coverage, health literacy, roles and responsibilities, financial, and transportation. The primary barrier in treating rural stroke survivors identified by OTs was lack of appropriate clinic equipment for neuro-specific interventions.

**Conclusions:**

Further research is needed in a larger sample size to explore the extent to which therapist-perceived barriers agree with the stroke survivor's perspective and facilitate solutions to overcome them.

## Introduction

Historically, individuals residing in rural areas, currently about 46 million people (U. S. Department of Health Human Services, 2024), have experienced inequities in regard to healthcare access (Hayes et al., 2025) as well as negative stigmatization through media portrayals, such as being inadequate or primitive compared to urban counterparts, and by exclusion or underrepresentation in policy (Malatzky and Couch, 2023). Individuals living in rural areas experience an increased rate of chronic disease, lower health literacy, and geographical barriers to accessing healthcare, which contribute to increased incidence of stroke (Georgakakos et al., 2022). Rehabilitation utilization and standards of care for post-acute stroke survivors vary significantly especially when comparing rural and urban areas within the United States (Danzl et al., 2016; Doherty et al., 2021; Jia et al., 2012; Kitzman et al., 2016). Rural stroke survivors are less likely to receive post-stroke rehabilitation than their urban counterparts (Jia et al., 2012) and many non-urban locales lack specialized stroke rehabilitation outpatient therapy clinics (Doherty et al., 2021). Further, rural stroke survivors may not receive secondary prevention education or information on resources that may be available to them post-stroke (Kitzman et al., 2016) and may not seek out resources on their own due to limited technology access or skills (Danzl et al., 2016). Factors underlying this apparent disparity in rehabilitation and prevention experienced by rural stroke survivors need further definition.

Due to decreased functional levels, impairments, and complications after stroke, post-acute occupational therapy (OT) is commonly needed after hospitalization. Post-acute OT improves functional outcomes, reduces impairment, and improves quality of life (Kimura et al., 2022; Shahid et al., 2023). General healthcare barriers experienced by those in rural areas have been identified and may provide clues to barriers to post-stroke rehabilitation. Those with lower incomes may not have access to a vehicle to make follow up appointments, leading to reliance on friends/family for transportation (Kingston et al., 2015; Solovieva and Walls, 2015; Syed et al., 2013). For stroke survivors, transportation challenges could be exacerbated by residual stroke symptoms, such as hemiparesis, limiting the ability to get in and out of a vehicle when transportation is available. Systemic infrastructure in rural areas limits public transportation, including ride share services (Kingston et al., 2015; Solovieva and Walls, 2015; Syed et al., 2013). When combined with the distance required to travel to specialty healthcare services, the ability to make appointments can be impacted (Kingston et al., 2015; Solovieva and Walls, 2015; Syed et al., 2013). Other reported barriers to general healthcare access in rural areas include lacking systemic informational systems regarding available community healthcare and social services, absent or minimal healthcare services in rural areas, inadequate insurance, limited financial resources, and distrust or lack of confidence in healthcare providers (Solovieva and Walls, 2015). The lack of knowledge of resources and access to services can impact the timeliness of beginning rehabilitation after a stroke. Further, follow-through with referrals to post-acute care can be challenging as rural stroke survivors may not be able or willing to travel longer distances for follow up care (Gregory et al., 2010), which is frequently required for rural populations seeking specialty services (Tran et al., 2024). Beyond these few studies, there is limited research to describe the barriers that are specific to post-acute therapy access in rural settings, with no studies to date specifically exploring barriers to outpatient OT access for rural populations.

Prior research studies on barriers to rural healthcare access as a whole were conducted more than a decade ago and may not be applicable in today's healthcare and reimbursement structure (Chan et al., 2009; Freburger et al., 2011; Jia et al., 2012). A gap analysis survey is a warranted next step to better define barriers in OT access and utilization for rural stroke survivors. Therapist insights into this gap in knowledge can be valuable but have been understudied. The purpose of the present study was to provide updated evidence regarding barriers to accessing post-stroke outpatient OT from the perspective of occupational therapists (OTs).

This project aimed to identify barriers in outpatient OT access and utilization for stroke survivors in the rural US by surveying OTs who work with rural stroke survivors. The following research questions were investigated: (1) *What barriers, if any, do OTs identify to outpatient OT access for stroke survivors in rural United States?* (2) *What barriers, if any, do outpatient OTs report in treating rural stroke survivors?*

## Methods

This study was approved by the authors' local Institutional Review Boards (IRBs). The research team, consisting of the three authors, developed a gap analysis survey to identify barriers to OT access in rural areas as well as barriers in stroke specific training of therapists. The first author completed a literature review to identify general barriers to rural healthcare access and drafted the gap analysis survey; the remaining authors edited and assisted with finalization of the survey. The gap analysis survey was distributed to OTs throughout the United States *via* social media, the Certified Stroke Rehabilitation Specialist certification email list, the first author's institutional stroke rehabilitation professionals email listserv, as well as authors' personal and professional contacts in rural areas of the United States. Email and social media communication invited OTs who currently or previously provided therapy to stroke survivors who identified as residing in rural geographic areas to complete the survey. Emails and social media communications were encouraged to be shared within recipients' personal and professional networks.

### Gap analysis survey

The gap analysis survey was developed based upon prior research involving rural healthcare access and utilization (Bayley et al., 2012; Dew et al., 2013; Freburger et al., 2011; Georgakakos et al., 2022; Juckett et al., 2020; Kingston et al., 2015; Morton et al., 2022; Salbach et al., 2007; Solovieva and Walls, 2015; Syed et al., 2013). The survey gathered information on therapist demographics, OT practice characteristics, potential barriers to outpatient OT access for rural stroke survivors (ranked on a four-point Likert scale: strongly disagree, disagree, agree, and strongly agree), and potential barriers to stroke specific training for rural practice OTs (ranked on a four-point Likert scale: strongly disagree, disagree, agree, and strongly agree). Open text was allowed to include potential supports for access and training as well as for any other general comments. Example gap analysis survey questions are presented in Figure 1.

**Figure 1:**
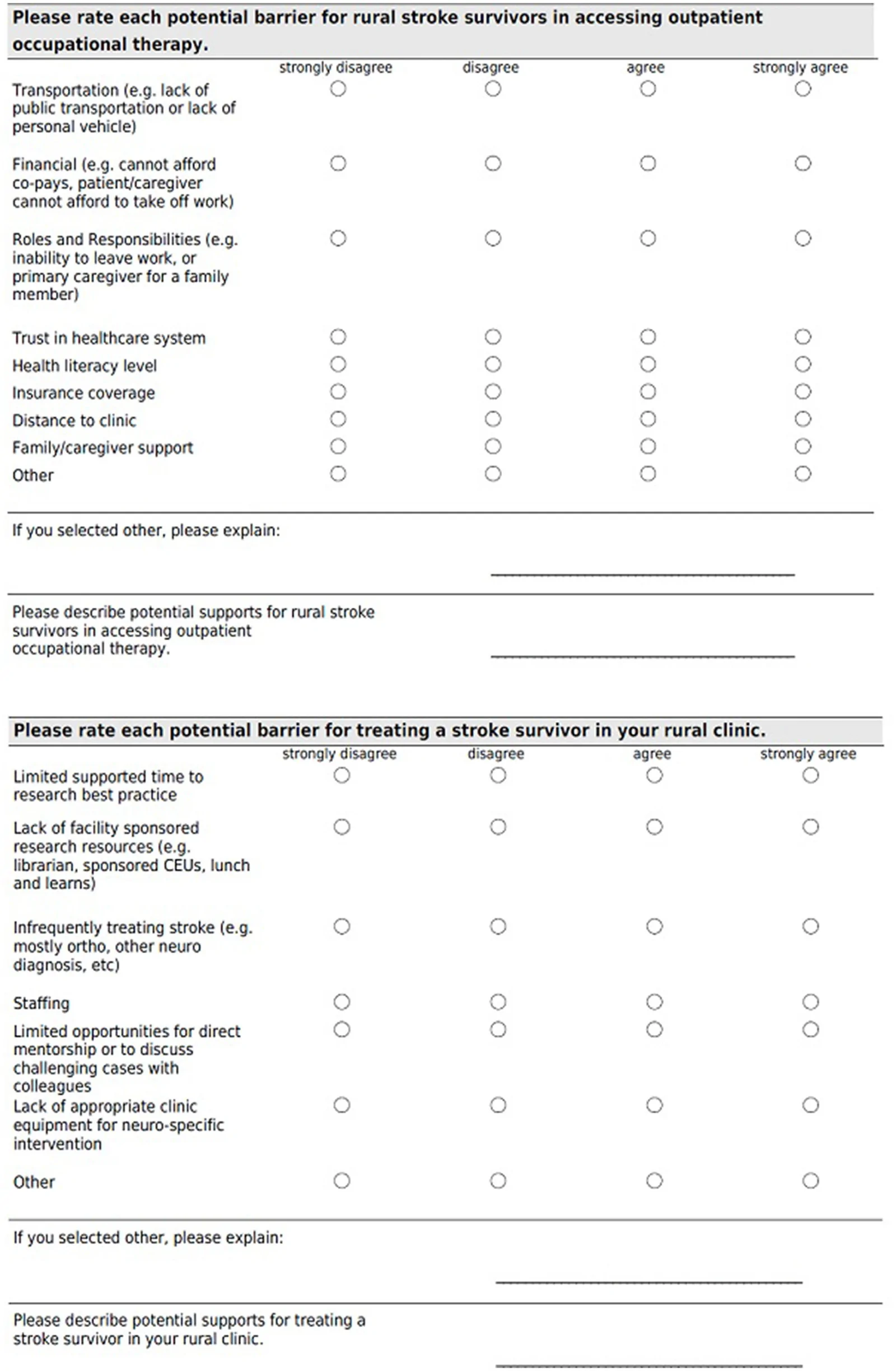
Example questions from gap analysis survey.

The gap analysis survey was designed within a REDCap database. A hyperlink and QR code were included within the recruitment tools. Upon accessing the hyperlink or QR code, respondents were directed to the invitation to participate within the REDCap database. Following acknowledgment of the invitation to participate, respondents were automatically directed to the gap analysis survey. The invitation to participate and the survey were available from February 2024 to September 2024.

### Data analysis

Due to the small number of respondents, Likert scale results were grouped into two categories to determine agreement or disagreement. Results were analyzed using descriptive statistics. Planned descriptive statistics included percentages, mean, median, and standard deviation where applicable. Open comments on the survey were summarized in the results section.

## Results

The survey was emailed to 1,072 individuals, across multiple email lists, and shared on four social media platforms. As the email lists included multiple disciplines involved in stroke rehabilitation, in both rural and urban areas of the U.S., it is unclear how many eligible OTs received invitation to complete the survey. Twenty-six OTs across 15 states completed the survey. The average age of respondents was 36.7 years with an average of 10.5 years in practice. Most respondents were Master's level educated (76.9%) and treated fewer than 10 stroke patients each week (69.2%). Detailed demographics are in Table 1.

**Table 1:** Therapist demographics.

Demographics	Results
Age (mean = 36.65, median = 35, SD = ±6.41) n = (%)	
20–29	2 (7.69)
30–39	18 (69.23)
40–49	5 (19.23)
50–59	1 (3.85)
Education	
Master's	20 (76.92)
eOTD	4 (15.38)
pOTD	2 (7.69)
Years in practice (mean = 10.54, median = 8, SD = ±6.83)	
1–9	14 (53.85)
10–19	9 (34.62)
20–29	3 (11.54)
Stroke patients per week (mean = 7, median = 3.5, SD = ±9.01)	
0–9	18 (69.23)
10–19	2 (7.69)
20–29	2 (7.69)
30+	2 (7.69)
Missing	2 (7.69)
Number of OTs in clinic (mean = 4.28, median = 3, SD = ±3.62)	
0–9	21 (80.77)
10–19	4 (15.38)
Missing	1 (3.85)
Location	
Alabama	1 (3.85)
California	1 (3.85)
Colorado	1 (3.85)
Illinois	2 (7.69)
Indiana	1 (3.85)
Kansas	1 (3.85)
Maryland	1 (3.85)
Mississippi	1 (3.85)
North Carolina	3 (11.54)
Pennsylvania	2 (7.69)
Tennessee	1 (3.85)
Texas	4 (15.38)
Virginia	2 (7.69)
Washington	1 (3.85)
West Virginia	2 (7.69)

The most commonly agreed upon barriers to outpatient OT were: financial (e.g., cannot afford co-pays, patient/caregiver cannot afford to take off work; 100% of participants agreed), transportation (e.g., lack of public transportation or lack of personal vehicle; 100%), insurance coverage (underinsured or uninsured; 96%), distance to clinic (92%), health literacy level (80%), family/caregiver support (73%), and roles and responsibilities (e.g., inability to leave work, or primary caregiver for a family member; 73%). Stroke survivors' trust in the healthcare system was not viewed as a barrier by most respondents (42%). Barriers to outpatient OT are detailed in Figure 2.

**Figure 2:**
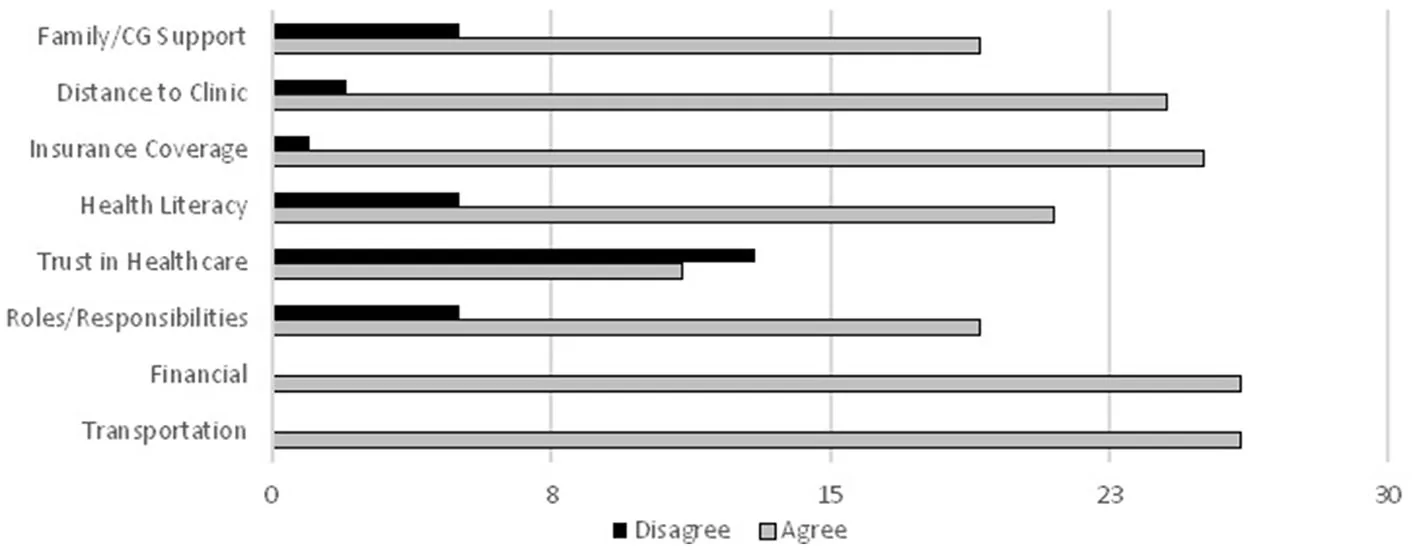
Barriers to outpatient occupational therapy access for rural stroke survivors.

Respondents included comments about barriers to outpatient OT for rural stroke survivors. These comments focused on transportation and distance to clinic barriers as primary barrier and included: “True rural clients don't live close to an outpatient clinic and must travel many miles to get to one”; “resources for stroke survivors are severely limited or poorly advertised/accessible. Or both”.

Therapists reported that barriers to their ability to treat rural stroke survivors were as follows (% agree): lack of appropriate clinic equipment for neuro-specific intervention (69%); limited opportunities for direct mentorship or to discuss challenging cases with colleagues (58%); lack of facility sponsored research resources [e.g., librarian, sponsored continuing education units (CEUs), lunch and learns; 54%]; limited supported time to research best practice (54%), staffing (inadequate or limited; 50%); and infrequently treating stroke (46%). Therapist-perceived barriers to treating rural stroke survivors are detailed in Figure 3. In response to a prompt to explain their responses, therapists elaborated on barriers to treating rural stroke survivors, which emphasized limitations in neuro-specific equipment. Equipment limitations included “funding for appropriate equipment for up-to-date technology” and “increasing access to appropriate equipment”.

**Figure 3:**
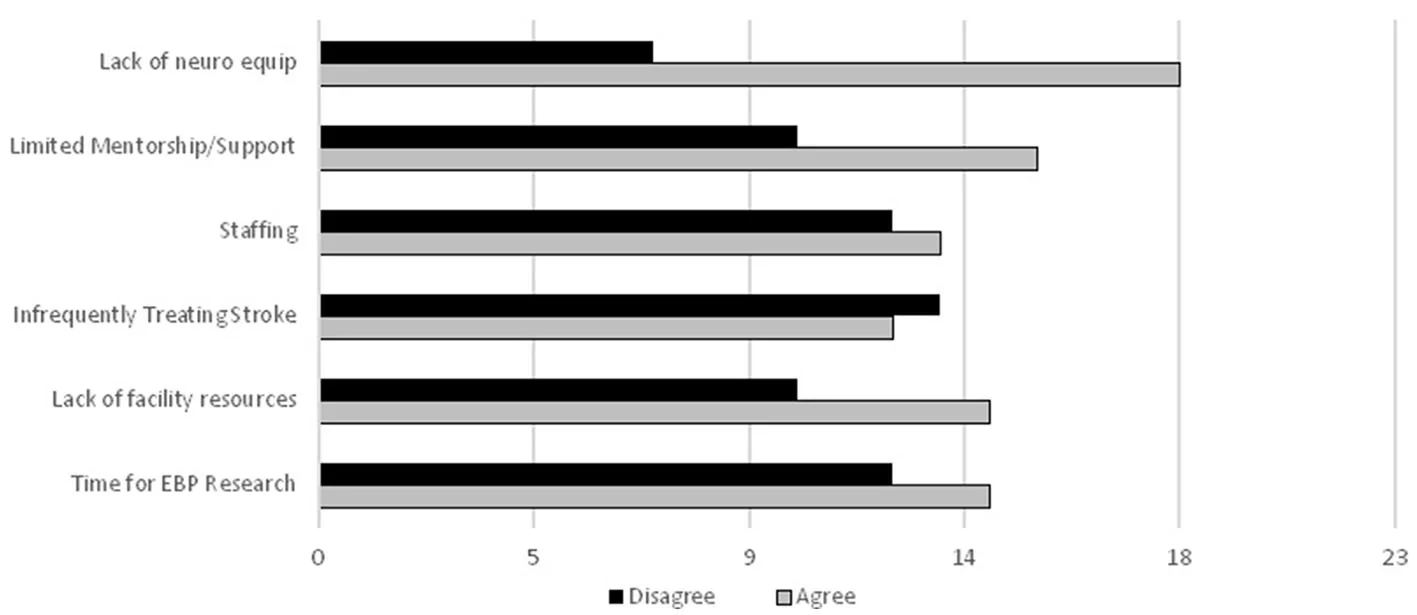
Barriers to treating rural stroke survivors among 26 respondents.

Many respondents also included recommendations in their comments for how to overcome the identified barriers for outpatient OT access for rural stroke survivors. Recommendations included increasing specialty rehabilitation access through telehealth/remote or mobile therapy sessions or at home self-management resources (such as video trainings, education), catering to caregiver needs by increasing time for caregiver training while in the hospital or increasing caregiver support outside of the hospital (such as support groups), addressing transportation needs through clinic, insurance, or charity funded transportation, and addition of outpatient case managers as part of multidisciplinary team. All comments are included in Table 2 and organized as facility, community, or systemic level barriers or supports.

**Table 2:** Open comments on gap analysis survey.

Category	Comments
[Barriers for stroke survivors] If you selected other, please explain:	
Facility level	Lack of education, resources and advocacy at acute/sub-acute stage of rehabilitation—at hospital when initial injury occurs, or in inpatient rehab.
	The significant amount of time and difficulty of dressing/transferring/transporting a stroke patient with excessive disability.
Systemic level	Distance to QUALITY outpatient stroke care as most outpatient rural clinics do not have certified specialists and patients often are encouraged to drive into larger cities to access better care for improved outcomes which is even more taxing on their support system and the patients (exhaustion from working hard mentally and physically and then a long drive)
Please describe potential supports for rural stroke survivors in accessing outpatient occupational therapy.	
Facility level	Telehealth, virtual reality
	Clinic transportation, Committee on Aging
	An outpatient neuro rehabilitation clinic
	Transportation options, additional OP case managers
Community level	Caregiver and family support.
	Increasing training and comfort on accessing *Via*/transportation services for those with disability. They are available in our town but comfort level and knowledge on access is barrier.
	Strong community and church ties. Ability to perform purposeful activity at home as part of a home exercise program
Systemic level	Better transportation and resources closer, especially lower level patients who need specialized transportation needs.
	Greater reliability on transportation through insurance (medicaid transport is unreliable)
	In our area, there are several options for OP neuro rehab so stroke survivors usually don't have to travel far
	I think it is not feasible for many to travel a long distance, so outpatient services at home would be best. This could be done after Home Health services stop. Telehealth options would be good as well if therapists aren't available in their area. Another option would be videos that the patient and caregiver could access at any time since it is hard for them to remember what is done during therapy.
	Public transportation options, community support groups, caregivers
	Virtual, web-based system to locate resources, low cost OOP resources
	Financial support for gas money, HEP items, etc and transport assistance
	Increasing Mobile outpatient services that provide in-home outpatient therapy for those with transportation/caregiver barriers
	Other transportation services outside of Metro, CRS benefits to pay for therapy, RSVP free therapy clinic
	Those with Medicaid, those with services at the center for excellence in disabilities (CED) in Morgantown, WV
[Barriers for therapists] If you selected other, please explain:	
Facility level	Limited environmental setup to address global occupational therapy goals and plan of care, and maximize patient gains.
	Budget cuts that no longer support additional certifications in specialties (comes out of therapist's pocket).
	Doubling and tripling of patients in outpatient when these patients often need hands-on and one-on-one treatment.
Please describe potential supports for treating a stroke survivor in your rural clinic	
Facility level/community level	Thankfully, we have state of the art equipment and technology to utilize. journal clubs, skills labs, encouragement for ongoing education.
	Organizational support for providing neuro rehab OT.
	Clinic is on campus with a hospital designated as a comprehensive stroke center
	Groups, peer group
	Clinic paying for CEUs additional clinic neuro-specific equipment- Saebo, omnicycle, trial splints/slings, diathermy, K-tape, high/low mat, trial DME
	My rural clinic has 1:1 sessions with opportunity for consistent therapist scheduling due to lower census.
	We have 2 OTRs in my clinic that are certified stroke rehabilitation specialists
	Increasing access to appropriate equipment. As a mobile outpatient therapist, my equipment is purchased out of pocket.
	Inservice, CEU opportunities, specialized speakers, time off for CEUs, mentorship
	Treating members of my community
Systemic level	Funding for appropriate equipment for up to date technology.
	We have good supports in place, majority of barriers are socioeconomic, transportation, and multiple co-morbidities affecting ability to come to clinic
Do you have any other comments or thoughts?	
Facility level	Patients have a significant lack of awareness and understanding of their condition or the aftermath by the time they receive outpatient OT. This contributes to delays in acquiring equipment, orthoses, understanding life changes that need to be made immediately, as well as managing musculoskeletal involvement that typically occurs with most strokes. Potential benefits of rehab are diminished greatly as more time passes since initial injury by the time they get to outpatient rehab. One of the biggest things I see is the lack of addressing of a hemiplegic arm by any rehab professionals in the acute or inpatient phase.
Systemic level	In my past experience, true rural clients don't live close to an outpatient clinic and must travel many miles to get to one. I think a much better option for these patients is in home services if available or telehealth. In addition, I think if they could also be supplied with some type of video package that they could access at any time with instruction on exercises and various rehab topics would be helpful as much of what is taught in rehab is not retained. Abundant caregiver training while in inpatient or at the hospital would be of utmost importance since the caregiver may be the primary person helping the stroke patient with therapy. I think we have to move beyond thinking that the therapist has to be present to do therapy because honestly these patients need to be doing activities daily not just a couple of times a week during therapy, and if they can only do activities/exercises when a therapist is present, how does that help them when they stop therapy?
	It would be great to offer virtual appointments in which we could teach family members in the clinic and then follow up at home virtually to decrease need to come to the clinic as often if there are things they can work on at home. Having grants to cover training to rural communities to offer the best practice CEU opportunities for Stroke and Brain Injury.
	Resources for stroke survivors are severely limited or poorly advertised/accessible. Or both.

## Discussion

This study aimed to explore barriers to outpatient OT access for stroke survivors, as well as perceived barriers to treating rural stroke survivors from the perspective of OTs. Results showed unanimous agreement that transportation and financial factors were both barriers for rural stroke survivors in accessing outpatient OT services. These results concord with rural populations' access to general healthcare services (Kingston et al., 2015; Solovieva and Walls, 2015; Syed et al., 2013). Other frequently agreed upon barriers to accessing outpatient OT services also aligned with the literature on barriers to general healthcare access: family/caregiver support, distance to clinic, insurance coverage, healthcare literacy, and roles and responsibilities (Dew et al., 2013; Kingston et al., 2015; Solovieva and Walls, 2015; Syed et al., 2013).

The most common barrier to treating rural stroke survivors, reported by more than 60% of respondents, was lack of appropriate equipment. Evidence- based neuro-specific therapy equipment may include high tech items, such as robotics, neuromuscular electrical stimulation, or functional electrical stimulation bikes, but may also include important low to no tech items (Hildebrand et al., 2023; Winstein et al., 2016). For example, having an appropriately sized therapy plinth is necessary as therapists may utilize neuro-facilitatory positions (quadruped) with stroke survivors that require the therapist to be on the mat with their patients. Single plinth therapy tables will not safely accommodate this. Other no-tech items may include hand paddles, air casts, or mobile arm supports that assist with positioning a hypertonic upper extremity for functional tasks or interventions to support occupations. While OTs are known for their creativity in developing interventions and intervention tools, access to neuro-specific equipment is imperative when treating this population to ensure outcomes are being maximized (Juckett et al., 2020). Without access to appropriate equipment for the specialized needs of stroke survivors, equitable rehabilitation cannot be achieved for this already vulnerable population.

Over half of respondents reported limitations in their treatment of rural stroke survivors related to mentorship and facility support to access best practice research. While these results are concerning, they do align with prior evidence-based practice research amongst rehabilitation therapists. Krueger et al. (2020) surveyed 578 OTs and found that 42% of respondents reported having no supported time (zero minutes per week) to engage in evidence-based practice activities, such as searching for research or guidelines relevant to their patient(s), critically appraising research, and discussing current research. Considering that many non-urban geographic areas lack specialized stroke rehabilitation outpatient therapies (Doherty et al., 2021), facility support to access best practice research and continuing education could make a significant difference in post-acute rehabilitation for rural stroke survivors.

While barriers to treating rural stroke survivors were reported, respondents also offered valuable comments on how to combat the identified barriers. The overarching themes of the comments were to offer telehealth sessions, provide better in-home resources (such as self-led video training), increasing support for caregivers (both in hospital and in the community), and offer funding availability for transportation. Telerehabilitation, as well as self- and caregiver-led rehabilitation, has been extensively explored in stroke research and may be a feasible option to improve access to services (Anderson et al., 2022; Banbury et al., 2018; Chen et al., 2020; Cramer et al., 2019; Dodakian et al., 2017; Eng and Pastva, 2022; Handelzalts et al., 2021; Laver et al., 2020). Current Procedural Terminology (CPT) codes for telehealth therapy services continue to have provisional approval through Centers for Medicare and Medicaid Services (CMS) in the United States (CMS.gov., 2024). Telerehabilitation, self-, and caregiver-led rehabilitation, may also offer lower cost rehabilitation options for un- and underinsured rural populations, providing much needed resources to populations who may otherwise receive no care at all, reducing healthcare costs due to preventable secondary complications such as falls, skin breakdown, and contractures. An example of a low-cost, hybrid stroke telerehabilitation program, utilizing telephone calls and text messages to support limited in-person sessions, showed improvements in physical function, participation, and quality of life immediately after the intervention, but without sustained effect 6 months later (Saywell et al., 2021). Further exploration into traditional and hybrid telerehabilitation, self-, and caregiver-led rehabilitation programs that provide longitudinal support *via* low-cost technologies, such as cell phones, could be beneficial. Caregiver burden has also been highly explored, with increased support for caregivers at all stages of stroke recovery being a very important factor to prevent burnout (Chin et al., 2022; Mack and Hildebrand, 2023; Qureshi et al., 2023). While a thorough discussion on potential solutions to the identified barriers is outside of the scope of this paper, the authors do agree that future research that supports rural stroke survivors in accessing services they need is pertinent for progression of care.

Solovieva and Walls (2015) surveyed rural traumatic brain injury survivors and their families in West Virginia, United States, and found that many respondents reported difficulty finding specialty care, a lack of advocacy and support, as well as a lack of confidence in their healthcare provider's professional skills. Within the current study, trust in the healthcare system was not viewed by most respondents as a barrier to patients' access to outpatient OT services. The disagreement among respondents on this potential barrier may be related to multiple factors. First, OTs are holistic practitioners with an emphasis on function and return to occupation (meaningful, daily life activities); these qualities may assist OTs with establishing rapport, facilitating support, and, thus, increasing trust. However, the disagreement may also be related to the lens of the therapist-respondents as previous research reporting trust in the healthcare system as a barrier was identified from people with lived experience, not healthcare providers. Respondents may have only interacted with stroke survivors who trust the healthcare system enough to receive care, vs. those who never seek follow-up rehabilitation for reasons which may include a lack of trust in the system. Further research is important to explore barriers and supports through the lens of those with lived experience, including stroke survivors and their caregivers.

A major limitation of this study was the sample size. However, we acknowledge other limitations. The gap analysis survey was developed by the authors to gather preliminary data and has not been extensively tested. In addition, the respondent rate was low. A longer window for survey completion may have resulted in a greater number of respondents. Despite these limitations, respondents were from fifteen different states throughout the United States. Another limitation is that the study design limited perspectives from other stakeholders in stroke rehabilitation, most importantly the stroke survivor and their caregivers; barriers identified by therapists may not be congruent with those identified by other stakeholders. While therapists have a unique view of barriers to serving rural stroke survivors, their view may be biased based on treating those that have trust within the healthcare system, and their experiences may not include interactions with stroke survivors who are not engaging with the healthcare system for assorted reasons. In addition, surveys innately are at risk for non-response bias. For this survey, therapists who were eligible but chose not to respond may have differing opinions than those who did respond. It is the long-term goal of the first author to expand this work further, gather more data on barriers from a diverse group of stakeholders including rural stroke survivors and caregivers, and develop tools to overcome the barriers both in access and in OT practice. In future work, rural community engagement techniques, such as targeted recruitment within rural community hubs (places of worship, senior centers, primary care physician offices, rural therapy clinics, etc.) will be utilized to expand the discussion of perceived barriers and supports of rural stroke rehabilitation to include key stakeholders.

## Conclusion

This study explored the following questions: (1) *What barriers, if any, do OTs identify to outpatient OT access for stroke survivors in rural United States?* (2) *What barriers, if any, do outpatient OTs report in treating rural stroke survivors?*. Results indicated that finances, transportation, insurance coverage, distance to the clinic, health literacy level, family/caregiver support, and roles/responsibilities are barriers to rural populations' access to outpatient OT. The main barrier that OTs reported that impacted their ability to treat rural stroke survivors was access to appropriate neuro-specific equipment in their clinic. Future research should identify barriers to post-acute stroke rehabilitation access from the perspective of stroke survivors as well as other allied health professionals, to determine agreement between stakeholders and facilitate intervention planning to combat the identified barriers.

## Data Availability

The raw data supporting the conclusions of this article will be made available by the authors, without undue reservation.
